# EcoDetect-YOLO: A Lightweight, High-Generalization Methodology for Real-Time Detection of Domestic Waste Exposure in Intricate Environmental Landscapes

**DOI:** 10.3390/s24144666

**Published:** 2024-07-18

**Authors:** Shenlin Liu, Ruihan Chen, Minhua Ye, Jiawei Luo, Derong Yang, Ming Dai

**Affiliations:** 1 School of Mathematics and Computer, Guangdong Ocean University, Zhanjiang 524008, China; 202111672419@stu.gdou.edu.cn (S.L.); 202111621104@stu.gdou.edu.cn (R.C.); hahamiao@stu.gdou.edu.cn (J.L.);; 2 Artificial Intelligence Research Institute, International (Macau) Institute of Academic Research, Macau 999078, China; 3 College of Ocean Engineering and Energy, Guangdong Ocean University, Zhanjiang 524088, China

**Keywords:** domestic waste, intricate environmental landscapes, lightweight, P2, CBAM, BiFPN

## Abstract

In response to the challenges of accurate identification and localization of garbage in intricate urban street environments, this paper proposes EcoDetect-YOLO, a garbage exposure detection algorithm based on the YOLOv5s framework, utilizing an intricate environment waste exposure detection dataset constructed in this study. Initially, a convolutional block attention module (CBAM) is integrated between the second level of the feature pyramid etwork (P2) and the third level of the feature pyramid network (P3) layers to optimize the extraction of relevant garbage features while mitigating background noise. Subsequently, a P2 small-target detection head enhances the model’s efficacy in identifying small garbage targets. Lastly, a bidirectional feature pyramid network (BiFPN) is introduced to strengthen the model’s capability for deep feature fusion. Experimental results demonstrate EcoDetect-YOLO’s adaptability to urban environments and its superior small-target detection capabilities, effectively recognizing nine types of garbage, such as paper and plastic trash. Compared to the baseline YOLOv5s model, EcoDetect-YOLO achieved a 4.7% increase in mAP_0.5_, reaching 58.1%, with a compact model size of 15.7 MB and an FPS of 39.36. Notably, even in the presence of strong noise, the model maintained a mAP_0.5_ exceeding 50%, underscoring its robustness. In summary, EcoDetect-YOLO, as proposed in this paper, boasts high precision, efficiency, and compactness, rendering it suitable for deployment on mobile devices for real-time detection and management of urban garbage exposure, thereby advancing urban automation governance and digital economic development.

## 1. Introduction

With the advancement of the economy and the improvement of living standards, a substantial amount of domestic waste is inevitably generated in daily life. Studies indicate that over the next 30 years, global waste production is projected to increase from 2.01 billion tons annually in 2016 to 3.4 billion tons [1]. Hence, the importance of domestic waste management in reducing environmental pollution and decreasing the risk of disease transmission is self-evident [2]. Generally, waste components in urban environments and public spaces include paper, plastic, and metal, among others [3,4]. Among these, plastic waste is the most abundant globally, with a greater negative impact on the environment and health compared to other types of waste [5]. Numerous studies have demonstrated that improper domestic waste management can lead to severe adverse effects on local environments, including but not limited to creating unsightly landscapes, fostering bacterial growth, and even adversely affecting local tourism, thereby impacting urban economic development [6,7,8]. Despite the recognition of the serious consequences of waste on the environment and health, many cities and public spaces in both developing and developed countries still face significant challenges in waste management [9]. One of the major obstacles to advancing waste management is the widespread dispersion of domestic waste in urban areas, with the manual collection and processing of domestic waste incurring high labor costs and high rates of omission and posing risks to the health of inspection workers. Therefore, the development of a lightweight, highly generalized algorithm capable of the real-time monitoring of street community domestic waste exposure using embedded surveillance cameras is crucial for reducing manual inspection costs and advancing urban domestic waste automation management and digital economic development.

Object detection algorithms have been prevalent in computer vision long before the advent of deep learning, and currently, object detection has become a vital research direction in the field of computer vision. Traditional object detection algorithms are primarily based on image processing and machine learning algorithms. Classical methods involve manually extracting geometric, edge, and texture-level features using classic image classification algorithms [10,11,12,13], followed by the classification of images using classifiers [14]. However, these methods are characterized by complex processes, low efficiency, low accuracy, and poor generalization. The superior performance of deep learning has made it widely adopted in the field of computer vision [15,16]. With the continuous development of deep learning research and the improvement of GPU performance, convolutional neural networks (CNNs) have been widely applied in the fields of object detection and image classification. The convolutional layers in deep CNNs possess excellent feature extraction capabilities, representing hierarchical semantic information of different layers in a feature representation form without the need for other traditional feature extraction algorithms. Existing object detection networks can be divided into two categories: two stage and one stage. The former generates candidate regions first and then classifies and regresses them. The latter directly regresses bounding boxes and class labels. Among the two-stage algorithms is Faster R-CNN [17], which is a faster region-based convolutional neural network and a representative algorithm that combines region proposal networks [17] and Fast R-CNN [18]. Nowakowski and Pamuła [19] used Faster R-CNN for electronic waste detection, achieving an average accuracy exceeding 90%. While Faster R-CNN achieves high accuracy, it is slow and performs poorly on small objects. To address these issues, one-stage algorithms such as Single Shot MultiBox Detector (SSD) [20] and “You Only Look Once: Unified, Real-Time Object Detection (YOLO)” [21,22] have been developed, offering faster speed but lower accuracy compared to two-stage algorithms. YOLOv5 [23] has become the most popular due to its outstanding overall performance. Liu et al. [24] proposed a garbage detection model based on YOLOv5, achieving garbage detection in simple backgrounds. Patel et al. [25] compared the garbage detection capabilities of several detectors, finding YOLOv5 superior to others. Mao et al. [26] designed a single-object detector based on YOLOv3 combined with the Taiwan recycling garbage dataset. Li et al. [27] proposed a multi-model cascaded CNN based on SSD, YOLOv4, and Faster R-CNN to reduce false-positive predictions of domestic waste. However, these detectors were mainly evaluated on datasets with simple backgrounds and single objects with fixed shapes. The detection of domestic waste under surveillance cameras faces more challenges due to small target objects, complex backgrounds, overlapping objects, and diverse waste shapes. Existing garbage detection models have yet to overcome these challenges, leaving considerable room for improvement in detection accuracy and speed. Additionally, some of these detectors have a large number of parameters, potentially compromising real-time performance.

To meet the demand for data-driven evaluation benchmarks for deep detection networks, some research has focused on constructing image datasets for garbage recognition. TrashNet [28] was the first public garbage dataset, consisting of images captured in residential areas and at Stanford University with a simple white background. Corresponding image enhancements were achieved through techniques such as random rotation and brightness adjustment. The GINI [29] dataset contains 2561 abandoned images obtained through Bing search engine queries and other sources. TACO [30] is an open image dataset used for garbage classification, detection, and segmentation tasks using images captured in outdoor scenes. Panwar et al. [31] proposed the AquaTrash dataset, consisting of 369 manually annotated images covering four types of garbage. Although these datasets provide sample libraries and evaluation benchmarks for garbage detection, the available domestic waste image datasets are scarce due to their small scale, simple backgrounds, or specific scene, and are non-open sources. As a result, these datasets are entirely inadequate for the application of domestic waste detection tasks under surveillance cameras as presented in this paper.

To address the current issues in domestic waste detection research, this paper proposes a lightweight, highly generalized, real-time domestic waste detection algorithm applicable to surveillance cameras, named EcoDetect-YOLO. Furthermore, a large dataset of domestic waste exposure detection under surveillance cameras was collected for training the model proposed in this study. The main contributions of this study are summarized as follows:

(1) Constructed a surveillance camera-based domestic waste exposure detection dataset comprising 3995 images capturing nine detection target types including paper trash, plastic trash, etc.
(2) Introduced a convolutional block attention module (CBAM) between the P2 and P3 layers in the backbone layer to extract key features and suppress irrelevant information.
(3) Added a high-resolution feature map layer, P2, in the neck layer to retain finer details and position information, enhancing efficiency and accuracy in detecting small garbage targets.
(4) Introduced a bidirectional feature pyramid network (BiFPN) to improve the model’s capability for deep feature fusion by upgrading the FPN and PANet structures in the neck layer to BiFPN.
(5) Conducted extensive experiments to evaluate the proposed EcoDetect-YOLO method in this study, with the experimental results demonstrating that the proposed EcoDetect-YOLO method achieves state-of-the-art detection performance on the proposed intricate environment domestic waste exposure detection dataset.

The remainder of this paper is organized as follows: Section 2 introduces the relevant methods of the original YOLOv5s network, multi-scale feature fusion, and CBAM attention mechanism. Subsequently, Section 3 describes the dataset proposed in this paper and the EcoDetect-YOLO algorithm. Detailed analyses of the experimental process are presented in Section 4. Finally, Section 5 summarizes the work of this study.

## 2. Related Work

### 2.1. YOLOv5

**Figure 1:**
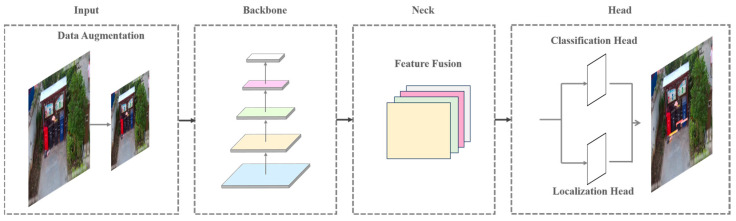
The detection architecture of YOLOv5 features input, backbone, neck, and head.

YOLOv5 is a one-stage object detection algorithm characterized by high computational efficiency and structural simplicity. Utilizing deep convolutional neural networks (CNNs), the model partitions the entire image into a series of grids, predicting the presence of objects in each grid along with their position, size, category, and other information. YOLOv5 primarily comprises four components: input, backbone, neck, and head, as illustrated in Figure 1.

Input: The input includes image data augmentation concatenation, the setting of three initial anchors, and the adaptive scaling of image size.

Backbone: The backbone network of YOLOv5 comprises CBS, C3, and SPPF. It transforms the raw input image into multiple layers of feature maps and extracts features for subsequent object detection. The CBS module, a fundamental module commonly used in convolutional neural networks, plays a role in feature extraction from input images, composed of Conv [32], BatchNorm [33], and SiLU [34]. Conv is the basic layer in CNNs, primarily used to extract local spatial information from input features. BatchNorm layer, attached after convolution layers, normalizes the distribution of feature values in the network. The SiLU activation function introduces a non-linear transformation capability to the neural network. While maintaining high accuracy, the C3 module significantly improves network computational efficiency, enhancing the speed and efficiency of object detection. The SPP module is a spatial pyramid pooling layer capable of handling inputs with different resolutions, achieving adaptive size output, increasing receptive fields, and extracting overall features of detection targets. YOLOv5 employs SPPF instead of SPP, reducing computation by half while achieving the same effect.

Neck: YOLOv5 adopts a combination of a feature pyramid network (FPN) [35] and path aggregation network (PANet) [36] in the neck. Compared to FPN, PANet combines bottom-up and top-down network structures, integrating feature maps of different scales. Complementary feature maps of different scales enhance detection performance.

Head: The head of YOLOv5 employs three detection heads to detect target objects and predict their categories and positions, corresponding to feature maps of 20 × 20, 40 × 40, and 80 × 80, outputting targets of different sizes [37].

**Figure 2:**
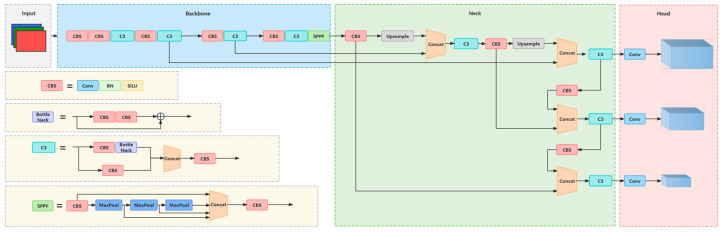
The overall network architecture of YOLOv5s.

In the YOLOv5 model, different models are categorized into YOLOv5s, YOLOv5m, YOLOv5l, and YOLOv5x according to different depths and widths [38], with corresponding increases in depth and width parameters resulting in improved model performance. However, this also leads to more complex network structures and slower detection speeds. Given the need for the real-time display of waste exposure and the desire to minimize operational memory, this study selected the YOLOv5s model, which has a relatively simple network structure and faster detection speed, as the baseline model. The overall network architecture of YOLOv5s is depicted in Figure 2.

### 2.2. Multi-Scale Feature Fusion

Multi-scale feature fusion [39] aims to enhance model performance by integrating features of different scales, which is particularly crucial in object detection tasks. Traditional neural networks typically use fixed-size filters or pooling operations when processing input images, which may lead to the loss of low-level details or high-level semantic information. Introducing multi-scale feature fusion into neural network architectures can address this issue. Various methods can achieve multi-scale feature fusion. One common approach is to concatenate or overlay feature maps of different scales, allowing the network to simultaneously utilize information from multiple scales for decision-making. Another method involves generating feature maps at different levels using a pyramid structure and merging them to enable the model to capture details and semantic information at different scales. Through multi-scale feature fusion, the model can better adapt to objects or scenes of different scales and sizes, enhancing its robustness and performance in complex scenarios. Multi-scale feature fusion is a significant problem in feature extraction, as features of different scales are crucial for various tasks. However, traditional top-down feature pyramid networks (FPNs) often fail to fully utilize features of different scales due to the limitations of unidirectional information flow, necessitating a more effective approach to address this issue [40]. The YOLOv5 algorithm employs the PANet network for feature fusion, which compared to FPN, incorporates a bottom-up path aggregation network to achieve bidirectional information flow [41]. However, the PANet network requires more parameters and computational resources, resulting in relatively slower speeds, making it less suitable for real-time object detection tasks. Additionally, although PANet adopts a bottom-up path aggregation network to enhance information flow, if low-level feature information is insufficient or partially lost, this method may lead to a decrease in detection accuracy. BiFPN, as a novel network structure for multi-scale feature fusion, builds upon FPN by adding a bottom-up feature path. Through bidirectional connections and the feature fusion of feature nodes in the feature pyramid network, BiFPN addresses the issue of insufficient utilization of features of different scales by traditional unidirectional FPNs, enhancing fusion accuracy and efficiency [42,43,44]. This study modifies the original FPN and PANet structures in the neck layer of YOLOv5 to BiFPN, thereby achieving more efficient multi-scale feature fusion.

### 2.3. Attention Mechanism

The attention mechanism [35] is employed to enhance neural networks’ focus on different parts of the input data, simulating the attention mechanism in the human visual system and allowing the network to selectively focus on information relevant to the current task. In traditional neural networks, each input is treated equally, and all features are simultaneously involved in computations. However, in many tasks, only a small portion of the input may be crucial for the output, and treating each input equally may lead to low algorithm efficiency and poor generalization [45]. Introducing the attention mechanism can effectively address this issue. The attention mechanism has achieved significant success in various deep learning tasks. For example, in image classification tasks, the attention mechanism can help the network automatically focus on regions of the image relevant to classification, thereby improving accuracy. In text summarization tasks, the attention mechanism enables the model to better focus on the most important content in the original text when generating summaries, resulting in more accurate and concise summaries [46]. To enhance focus on key regions, suppress unimportant background information, and improve detection accuracy, this study introduces the CBAM attention module. CBAM simultaneously considers spatial and channel dimensions, calculates attention weight coefficients through spatial attention modules and channel attention modules, and multiplies them with input feature maps to adaptively refine input features [47]. By adding CBAM attention modules between the feature extraction backbone network and the inference layer, the YOLOv5s algorithm can identify regions with high feature map weights and focus more on important features during inference.

## 3. Materials and Methods

### 3.1. Dataset

This study utilized a multi-objective dataset for detecting urban solid waste from surveillance cameras, primarily sourced from the China International Big Data Industry Expo in Guizhou Province, China. [48]. The surveillance videos were in MP4 format with frames of 1920 pixels in width and 1080 pixels in height and a frame rate of 30 frames per second. After manual selection from community road videos containing solid waste, individual image frames were extracted and processed for subsequent analysis.

**Figure 3:**
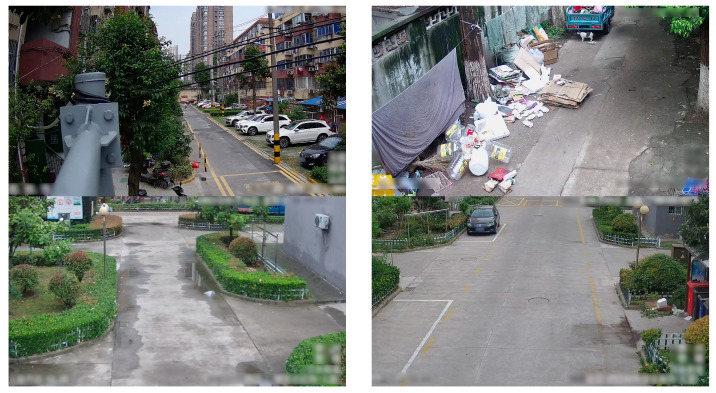
Example of the Multi-Target Life Garbage Exposure Image Dataset.

This dataset facilitates the identification of exposed urban solid waste in communities and advances digital ecological conservation efforts, offering crucial data support and industry benchmarks. The predominant backgrounds of the collected data are roads and community settings, featuring a variety of environmental conditions including daytime, nighttime, rainy, clear, and snowy weather, thereby providing diverse environmental contexts. However, the dataset includes images that are blurry, incomplete, or unidentifiable, which may degrade model training performance. To address this challenge, extensive manual curation was undertaken to identify and remove problematic images, resulting in a refined dataset comprising 3995 images, exemplified by Figure 3.

**Figure 4:**
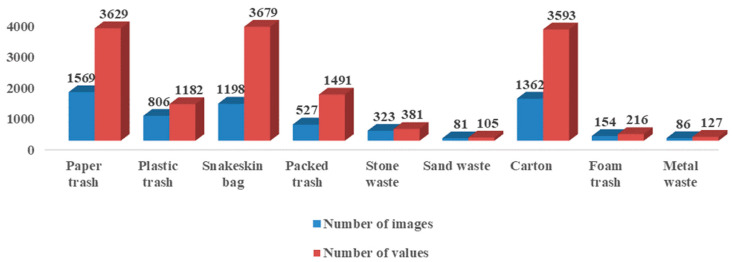
Dataset distribution: the blue bar chart represents the distribution of the number of images for each type of garbage; the red bar chart represents the distribution of the number of each type of garbage.

These images were annotated using the open-source image annotation tool LabelImg, including target type, center point, width, and height., including target type, center point, width, and height. The number of images and number of values corresponding to each category were obtained, as shown in Figure 4. There were nine types of detection targets: paper trash, plastic trash, snakeskin bags, packed trash, stone waste, sand waste, carton, foam trash, and metal waste. During the experimental process, the dataset was randomly divided into training and testing sets in an 8:2 ratio for the training and testing of the YOLOv5s model.

### 3.2. Focal Loss

This study addressed the notable issue of sample imbalance within the garbage dataset by employing the focal loss method [49] in the training process of the YOLOv5s model, which typically utilizes a composite loss function. The focal loss, defined by Equation (1), effectively mitigates sample imbalance as follows:

(1) $$FL\left(p,y\right)=f\left(x\right)=\left\{\begin{array}{c} -\alpha {\left(1-p\right)}^{\gamma }\log(p),\;\;if\;y=1\\ -(1-\alpha )p^{\gamma }\log(1-p),\;\;otherwise \end{array}\right..$$

In Equation (1), α is the weight controlling the balance between positive and negative samples, ranging from [0, 1]. The variable p represents the predicted probability of a sample, also ranging from [0, 1]. When p is close to 1 or 0, the features of such samples are more pronounced and easier to learn, and they are categorized as easy samples. Conversely, when p is around 0.5, the sample is considered difficult. The variable y indicates whether a sample is positive (*y* = 1) or negative; samples are typically classified as positive if p>0.5; otherwise, they are classified as negative. The parameter γ is the focusing parameter, where higher values increase the model’s focus on distinguishing between positive and negative samples.

Equation (1) also illustrates that within the positive sample set (p > 0.5) or within the negative sample set, higher values of p and lower values of p, respectively, result in smaller calculated values of *FL*(*p*,*y*). This implies that easier-to-learn samples contribute less to the loss function, thereby directing the model’s training focus toward more challenging samples. Simultaneously, the parameter α ensures equal treatment of positive and negative samples, preventing the model from overemphasizing high-quality samples during the training process.

This study successfully utilized the focal loss method to effectively address the sample imbalance problem, enhancing the robustness, performance, and stability of the model.

### 3.3. Dataset Preprocessing

To enhance the performance of the YOLOv5s model, this study implemented preprocessing steps recommended by official guidelines, employing standard methods such as resizing, normalization, and data augmentation. Detailed descriptions of the resizing, normalization, and data augmentation methods are provided below.

#### 3.3.1. Resizing

Given the varied dimensions of images in the household waste dataset, this study addressed potential sample imbalance by unifying the size of all images to a standard specification. The resizing process involves adjusting the matrix size of the images. Traditional scaling methods often result in image distortion; therefore, this study adopted an aspect-ratio-preserving method. The specific steps are as follows:

Step 1: Calculate the scaling ratio r.

(2) $$r=\min\left(\frac{W_{new}}{W},\frac{H_{new}}{H}\right).$$

Step 2: Compute the unpadded width and height.

(3) $$W_{unpad}=round\left(W\times r\right),$$

(4) $$H_{unpad}=round\left(H\times r\right),$$

In Equations (3) and (4), both $W_{unpad}$ and $H_{unpad}$ are integers.

Step 3: Calculate the required padding.

(5) $$dw=\left(W_{new}-W_{unpad}\right)mod\;Stride,$$

(6) $$dh=\left(H_{new}-H_{unpad}\right)mod\;Stride.$$

The padding ensures that dimensions are divisible by the stride, optimizing the receptive field. Stride represents the downsampling ratio per layer in the model, with a total stride of 32 in the original YOLOv5 model, requiring five downsampling steps.

Step 4: Calculate the padding boundaries.

Divide the fill evenly between the two sides.

(7) $${dw}^{\prime }=\frac{dw}{2},$$

(8) $${dh}^{\prime }=\frac{dh}{2}.$$

Calculate the amount of fill in four directions.

(9) $$\left\{\begin{array}{c} top=round\left({dh}^{\prime }-0.1\right)\\ botton=round\left({dh}^{\prime }+0.1\right)\\ left=round\left({dw}^{\prime }-0.1\right)\\ right=round\left({dw}^{\prime }+0.1\right) \end{array}\right..$$

Step 5: Use OpenCV’s copyMakeBorder() function to resize the images and add them to a 640 × 640 pixel gray background.

**Figure 5:**
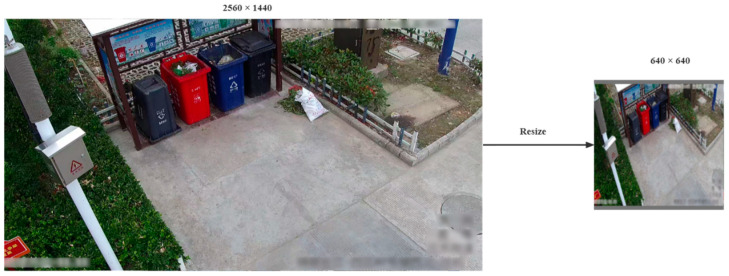
Results of adaptive scaling applied to original images.

The results are shown in Figure 5.

#### 3.3.2. Normalization

In the YOLOv5 model, batch normalization is typically employed to normalize each mini-batch of image data. The objective is to ensure a more stable and faster convergence during the training process, reduce the dependency on initial parameters, and enhance the model’s generalization capability.

Given a mini-batch of image data inputs $X=\left\{x_{1},x_{2},\ldots ,x_{m}\right\}$, where m denotes the size of the mini-batch, the specific implementation steps are as follows:

Step 1: Calculate the mean of the mini-batch.

(10) $$\mu_{B}=\frac{1}{m}\sum_{i=1}^{m}x_{i}.$$

Step 2: Calculate the variance of the mini-batch.

(11) $$\sigma_{B}=\frac{1}{m}\sum_{i=1}^{m}{\left(x_{i}-\mu_{B}\right)}^{2}.$$

Step 3: Normalize each input sample $x_{i}$.

(12) $$x_{i}^{\prime }=\frac{x_{i}-\mu_{B}}{\sqrt{{\sigma_{B}}^{2}+\varepsilon }},$$

In Equation (12), ε is a small constant added to prevent division by 0.

#### 3.3.3. Data Augmentation

**Figure 6:**
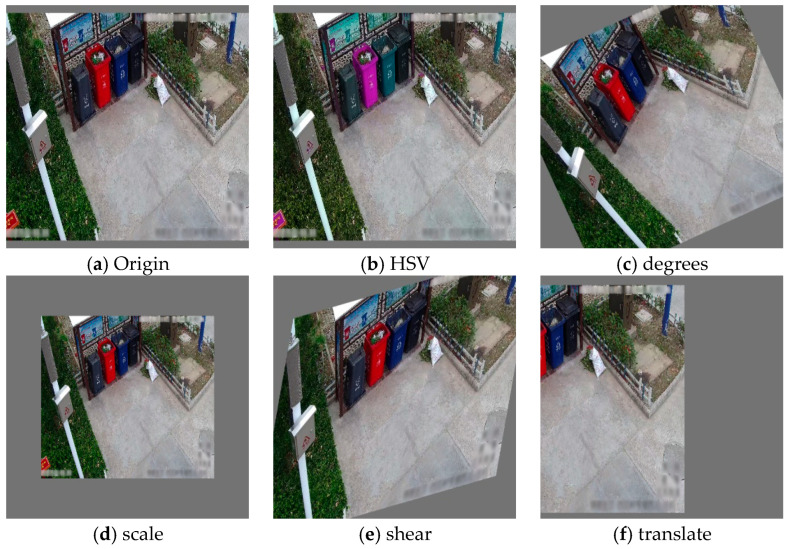
Results of HSV color-space augmentation and random perspective augmentation. (**a**) Original image, (**b**) image after HSV color-space augmentation, and (**c**–**f**) images after random perspective augmentation with adjustments in degrees, scale, shear, and translate, respectively.

Data augmentation refers to the technique of expanding the diversity of image samples in a dataset through non-repetitive region methods. This enhances the variety of data samples, crucially supporting the YOLOv5s model by incorporating methods such as HSV color-space augmentation, random perspective augmentation, mosaic augmentation, and MixUp augmentation:

As illustrated in Figure 6b, HSV color-space augmentation involves random alterations to the hue, saturation, and value dimensions of images. This allows the model to encounter a wider range of color variations during training, thereby enhancing its generalization capabilities under different lighting conditions and color changes.

As depicted in Figure 6c–f, random perspective augmentation introduces random perspective transformations in image processing. The parameters typically include the following:

1. Degrees: Specifies the angle range for perspective transformation, randomly chosen between −10° and +10°.
2. Scale: Defines the scaling factor range for transformation, randomly selected between 0.9 and 1.1.
3. Shear: Indicates the shear angle range for in-plane image skew, randomly chosen between −10° and +10°.
4. Translate: Specifies the range for image translation, usually expressed as a proportion of image width or height. A typical setting of translate = (0.1, 0.1) indicates that the image can be shifted horizontally or vertically within 10% of its width or height randomly.

Combining these parameters generates a diverse array of perspective-transformed images, enabling the model to adapt to various angles and viewpoints during training.

**Figure 7:**
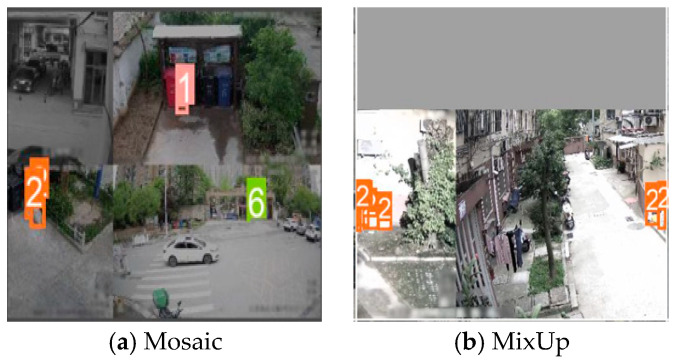
Results of mosaic augmentation and MixUp augmentation. (**a**) Image after mosaic augmentation and (**b**) image after MixUp augmentation.

As shown in Figure 7a, mosaic augmentation involves combining multiple images in specified proportions, followed by random cropping of the composite image. This technique simulates complex scenes with multiple objects, enhancing the model’s generalization and robustness to object location and size variations. The process includes the following steps:

1. Randomly select four images from the dataset and combine them into a mosaic. Each image’s position and size in the mosaic are random, with possible overlaps.
2. Randomly select a region from the generated mosaic and crop it as the training sample.
3. Adjust the positional information of sample labels within the cropped image to ensure accurate placement relative to the new image dimensions.

Illustrated in Figure 7b, MixUp augmentation blends two image samples to improve model generalization. The process involves the following steps:

1. Randomly select two images and their corresponding sample labels from the dataset.

2. Mix the images and labels in specified proportions to create a new MixUp image and label set, as described by the following equations:


(13) $$X_{Mix}=\lambda X_{1}+\left(1-\lambda \right)X_{2},$$

(14) $$y_{Mix}=\lambda y_{1}+\left(1-\lambda \right)y_{2},$$

where $X_{1}\;and\;X_{2}$ are the original images, $X_{Mix}$ is the generated MixUp image, $y_{1}$ and $y_{2}$ are the sample labels for the original images, and $y_{Mix}$ is the label information for the MixUp image.

### 3.4. Methods

#### 3.4.1. Convolutional Block Attention Module (CBAM)

In the realm of deep learning, the training and prediction processes of models necessitate the handling of extensive high-dimensional data, where the significance of individual features diverges. In such scenarios, an attention mechanism (AM) is indispensable to allocating heightened weights to pivotal features, thereby enabling deep learning models to concentrate more profoundly on these aspects [47]. The convolutional block attention module (CBAM), introduced by Sanghyun Woo in 2018, stands as a straightforward yet efficacious feedforward convolutional neural network attention module [50].

**Figure 8:**
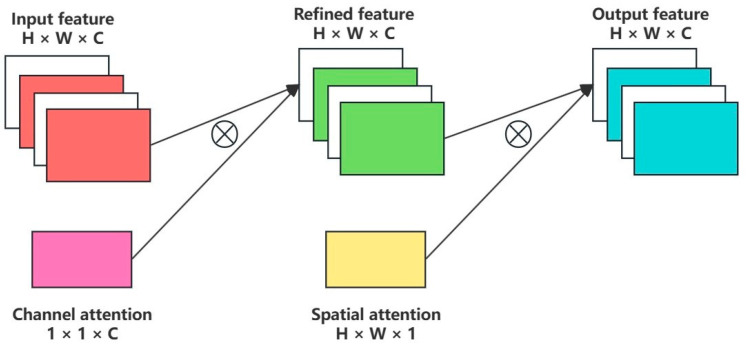
Structure diagram of the CBAM attention mechanism.

The CBAM integrates two distinct sub-modules, namely the channel attention module and the spatial attention module, to enhance the model’s understanding and processing capabilities of input features. Its structure is depicted in Figure 8.

**Figure 9:**
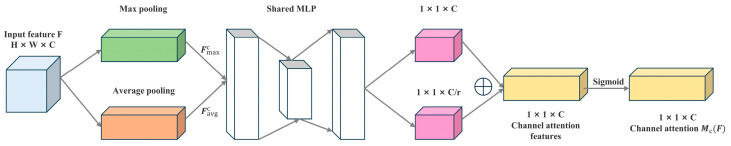
CBAM channel attention module.

The process flow of the channel attention module is illustrated in Figure 9. This module focuses on extracting the most critical attributes from the input fault features. It performs max pooling and average pooling operations on the input feature map F, yielding two sets of salient features, $F_{max}^{c}$ and $F_{avg}^{c}$, respectively. These features are subsequently processed by a shared multi-layer perception (MLP) through a series of perceptual layers, generating two unique feature vectors. These vectors are element-wise summed and then passed through a sigmoid activation function to produce the final channel attention feature M_c_ (F), which emphasizes the importance of specific channels. The computation of channel attention is represented by Equation (15).

(15) McF=σMLPAPF+MLPMPF                   =σW1W0Favgc+W0W1Fmaxc,

In Equation (15), AP and MP denote the average pooling and max pooling processes, respectively; σ represents the sigmoid activation function; and $W_{0}$ and $W_{1}$ are weights in the shared MLP.

After the channel attention feature map $M_{c}\left(F\right)$ is obtained from the channel attention module, it is multiplied with the input feature map F to yield the enhanced feature map F′, which serves as the input feature map for the spatial attention module. This computation is described by Equation (16).

(16) $$F^{\prime }=M_{c}\left(F\right)⊗F.$$

**Figure 10:**
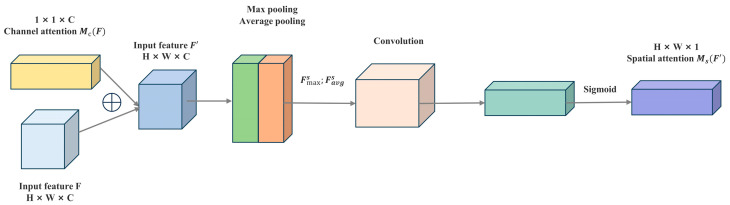
CBAM spatial attention module.

The process flow of the spatial attention module is depicted in Figure 10. This module shifts the focus to optimizing features from a spatial dimension. It receives the output F′ from the channel attention module as input and performs max pooling and average pooling operations to capture the spatial distribution of different features, denoted as $F_{max}^{s}$ and $F_{avg}^{s}$, respectively. By concatenating these pooling results and passing them through an additional convolution and sigmoid activation step, the module generates a weighted spatial feature map $M_{s}\left(F^{\prime }\right)$. The computation of spatial attention is given by Equation (17).

(17) MsF′=σf7×7([APF′;MPF′])     =σf7×7([Favgs;Fmaxs]),

In Equation (17), $f^{7\times 7}$ represents the convolution kernel size of the additional convolution layer.

The spatial feature map $M_{s}(F^{\prime })$ is then multiplied by the original input feature $F^{\prime }$ to obtain the final feature map $F^{\prime \prime }$. This feature map incorporates both channel and spatial attention information and is used for further processing in subsequent network layers. The computation is outlined in Equation (18).

(18) $$F^{\prime \prime }=M_{s}\left(F^{\prime }\right)⊗F^{\prime }.$$

In summary, CBAM, through these two complementary modules, effectively enhances the model’s attention to significant features across both channel and spatial dimensions, thereby augmenting the richness and accuracy of essential feature representation.

#### 3.4.2. Neck Network with Fusion of P2 Features

Small-object detection relies on deep semantic information and low-level detail information, but it is difficult for simple feature fusion methods to significantly improve its detection accuracy [51]. During small-object detection, due to the small size of the small-object samples in the image and the need for multiple downsampling in the backbone layer of the YOLOv5s model, the feature information of the obtained samples is reduced. When passing through the neck layer, the model finds it difficult to learn the feature information of small-object samples. In the three detectors of the head layer, smaller targets cannot be detected, leading to missed detections of small-object samples in the detection results. Therefore, to better adapt the model to small-object detection, a small-object detector is added to the head to fuse deep feature maps with shallow feature maps for detection, thereby effectively improving the accuracy of the model in detecting small-object samples.

The original YOLOv5s model network structure has three detectors. If the input image size is 640*640, after multiple downsampling in the backbone layer and feature fusion in the neck layer, detection feature maps of 20 × 20, 40 × 40, and 80 × 80 can be obtained, corresponding to targets larger than 32 × 32, 16 × 16, and 8 × 8, respectively. If there are samples smaller than 8 × 8 in the input image, the YOLOv5s model finds it difficult to detect these samples, resulting in missed detections.

**Figure 11:**
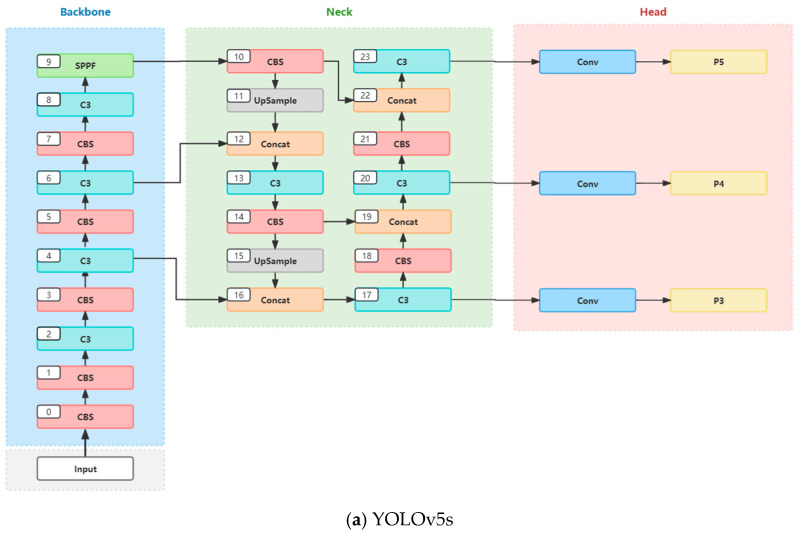

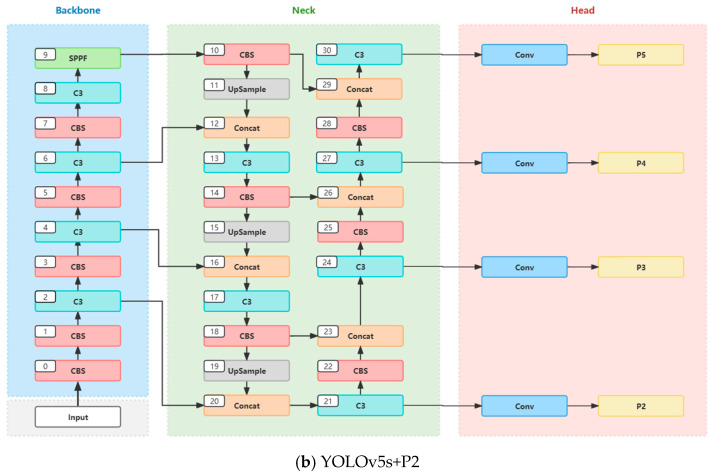
Network architecture diagram of the model. (**a**) YOLOv5s model. (**b**) YOLOv5s+P2 model.

To address the aforementioned challenges, this study introduces the P2 small-object detection layer into the YOLOv5s model. The primary operational flow is depicted in Figure 11. In the original YOLOv5s model (Figure 11a), following the Concat operation of the neck’s 16th layer, the result is directly forwarded to the 17th layer for C3 processing. In contrast, in the modified YOLOv5s+P2 model (Figure 11b), the output from the 16th layer after Concat, featuring 256 channels, is expanded downward. Due to the subsequent C3 operation in the 2nd layer resulting in a feature map of only 128 channels, an initial C3 (17th layer) and CBS (18th layer) operation is first performed. The resulting feature map, containing 128 channels, is then sent to the 19th layer for upsampling. At the 20th layer, the feature maps from the 2nd and 19th layers are concatenated, followed by C3 processing in the 21st layer, facilitating the output of small-object detection feature maps. Notably, the 17th layer in the original YOLOv5s framework is shifted to the 24th layer in the YOLOv5s+P2 model, thus augmenting the network framework by 7 layers of operations.

Ultimately, detection is performed using the head layer, resulting in detection feature maps of 20 × 20, 40 × 40, 80 × 80, and 160 × 160. The newly obtained 160 × 160 detection feature map enables the detection of samples larger than 4 × 4. With richer feature information of small objects in the 2nd layer’s feature map, the model enhances its adaptability for small-object detection during subsequent fusion processes. The overall network structure of the YOLOv5s model with the added P2 small-object detection layer is depicted in Figure 11.

**Figure 12:**
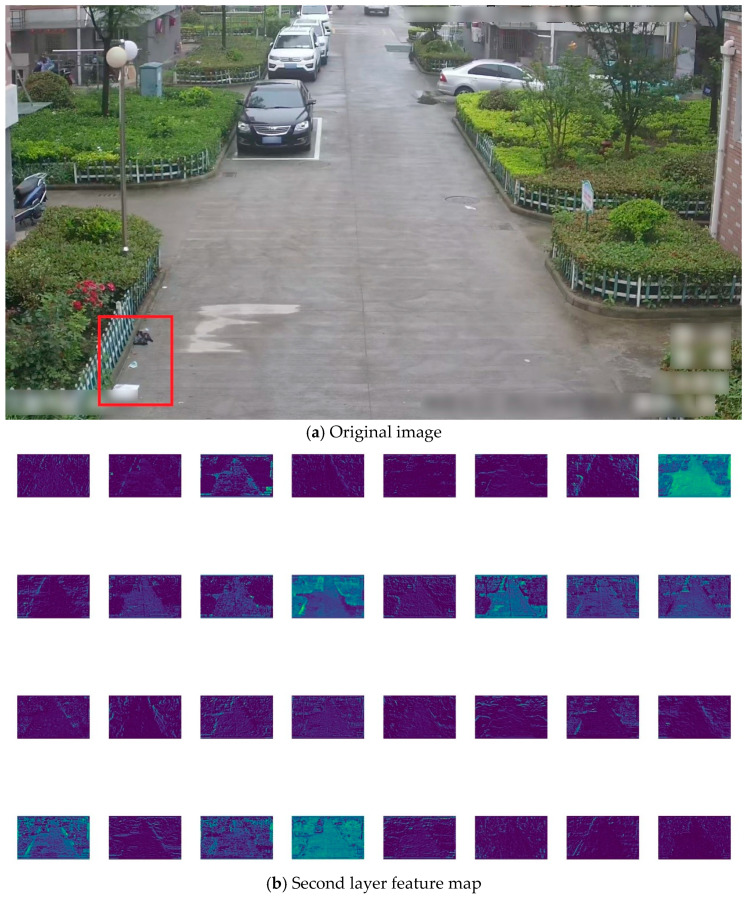

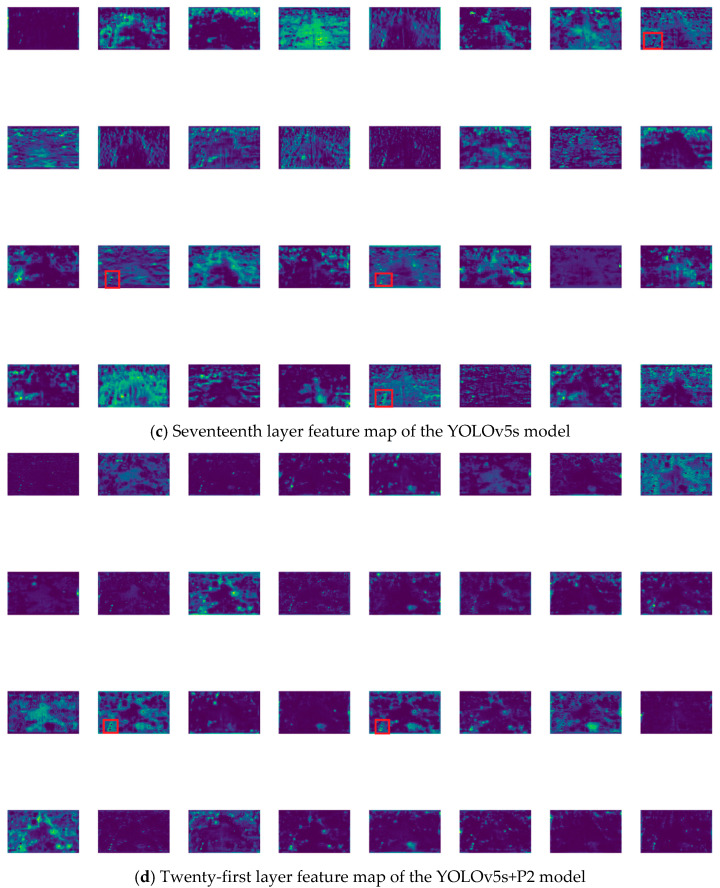
Feature maps of the YOLOv5s and YOLOv5s+P2 models. (**a**) Original image, (**b**) 2nd layer feature map, (**c**) 17th layer feature map of the YOLOv5s model, and (**d**) 21st layer feature map of the YOLOv5s+P2 model. The red boxes are used to mark the positions of the three samples.

To highlight the contribution of the added P2 small-object detection layer in the YOLOv5s model, Figure 12a presents the original image. Subsequently, feature maps are generated using pre-trained YOLOv5s and YOLOv5s+P2 models, respectively. The YOLOv5s model selects the feature map from the 17th layer (Figure 11a), resulting in what appears in Figure 12c, while the YOLOv5s+P2 model chooses the feature map from the 21st layer (Figure 11b), as shown in Figure 12d. Given that each model selects 256 feature maps, a subset of 32 maps is randomly chosen for convenience.

In the original image, three sample targets are enclosed within a red box, consisting of two larger and one smaller sample, with the black indicating plastic trash and the other paper trash. After multiple downsampling stages in the network model, the feature information of smaller samples gradually diminishes, particularly evident in the reduced feature information of the smaller sample in Figure 12c. Conversely, as illustrated in Figure 12d, the YOLOv5s+P2 model retains clear information about all three target samples even after multiple downsampling stages, attributed to the inclusion of feature map information from the 2nd layer (Figure 12b), which preserves numerous original image sample features.

**Figure 13:**
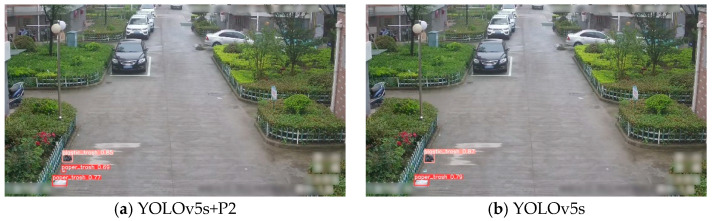
Testing results of the YOLOv5s+P2 and YOLOv5s models. (**a**) YOLOv5s+P2 model testing result. (**b**) YOLOv5s model testing result.

Testing YOLOv5s+P2 and YOLOv5s models on the original image yielded the results shown in Figure 13a,b, respectively. It is evident that the YOLOv5s model misses the smaller target among the three targets, whereas the YOLOv5s+P2 model successfully detects all targets.

#### 3.4.3. Bidirectional Feature Pyramid Network (BiFPN)

The FPN and PANet structures in the neck layer of the YOLOv5s model perform multi-scale feature fusion of input image features. The FPN structure transmits image semantic feature information from top to bottom, while the PANet structure transmits image positional feature information from bottom to top. Due to the presence of sample feature information with different resolutions in the input image, simple feature aggregation occurs during fusion, leading to an unreasonable contribution of features to the output.

To better address the above issues and improve the accuracy and efficiency of the model, a weighted bidirectional feature pyramid network (BiFPN) was proposed by Tan et al. from the Google AI research group, allowing for simple and rapid multi-scale feature fusion [52]. The BiFPN module utilizes a weighted feature fusion mechanism, as shown in Equations (19) and (20), enabling the model’s network structure to learn the importance of feature information at different resolutions in the input image. Simultaneously, it employs a fast normalization method, as shown in Equation (21).

(19) $$P_{i}^{td}=Conv\left(\frac{\omega_{1}\cdot P_{i}^{in}+\omega_{2}\cdot Resize\left(P_{i+1}^{in}\right)}{\omega_{1}+\omega_{2}+\varepsilon }\right),$$

(20) $$P_{i}^{out}=Conv\left(\frac{\omega_{1}^{\prime }\cdot P_{i}^{in}+\omega_{2}^{\prime }\cdot P_{i}^{td}+\omega_{3}^{\prime }\cdot Resize\left(P_{i-1}^{out}\right)}{\omega_{1}^{\prime }+\omega_{2}^{\prime }+\omega_{3}^{\prime }+\varepsilon }\right),$$

In Equations (19) and (20), *P_i_^in^* represents the input sample feature information of the i-th layer node, *P_i_^td^* represents the intermediate feature information of the top-down transmission path of the i-th layer, and *P_i_^out^* represents the output feature information of the bottom-up transmission path of the i-th layer. *Conv* represents the convolution operation, and *Resize* represents upsampling or downsampling operations.

(21) $$O=\sum_{i}\frac{\omega_{i}\cdot I_{i}}{\varepsilon +\sum_{j}\omega_{j}},$$

**Figure 14:**
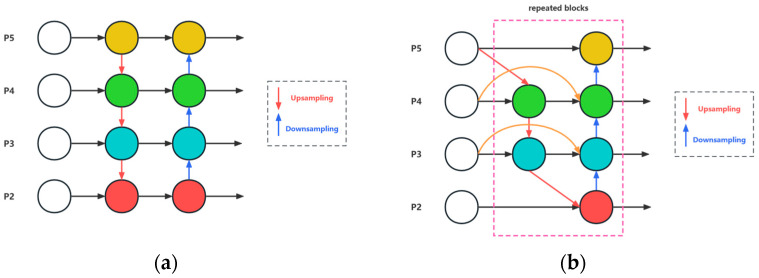
BiFPN network architecture diagram. (**a**) FPN and PANet structures in the YOLOv5s model after addition the P2 small-object detection layer. (**b**) BiFPN structure in the YOLOv5s model after addition of the P2 small-object detection layer.

In Equation (21), *O* represents the output value, *I_i_* represents the input value of the node, *ω_i_* represents the weight of the input node, and *j* represents the sum of all input nodes [53]. To ensure that the condition *ω_i_* ≥ 0 for each input node’s weight *ω_i_*, a ReLU activation function is applied to each *ω_i_*. Setting *ε* = 0.0001 avoids numerical instability. By using this method, the numerical range of each *ω_i_* is scaled to be between 0 and 1. Moreover, since the Softmax operation is not employed, the training speed and efficiency are improved. Figure 14 illustrates the BiFPN structure.

Nodes with only one input edge do not perform feature fusion and contribute less to the feature network aimed at fusing different features. Hence, these nodes are removed, simplifying the bidirectional network structure. For input nodes and output nodes at the same level, an additional edge is added between them to efficiently fuse higher-level features during multiple iterative superposition processes.

#### 3.4.4. Proposed EcoDetect-YOLO Model

Based on the advantages of the YOLOv5s model in small-object detection, an improved EcoDetect-YOLO model is proposed in this paper for detecting exposed garbage in general landfills and residential community road surfaces. The model underwent three main improvements: the introduction of the CBAM attention mechanism, P2 small-object detection head, and BiFPN structure.

**Figure 15:**
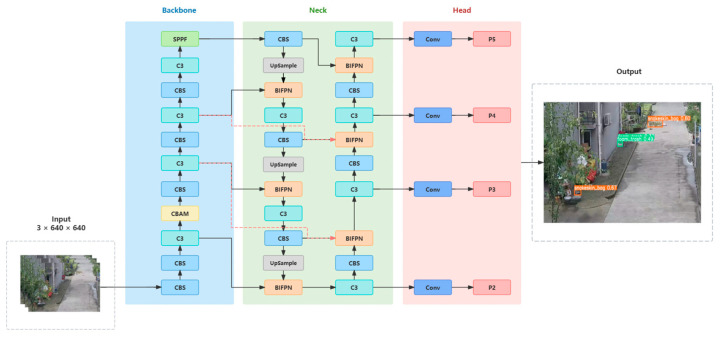
EcoDetect-YOLO overall network architecture.

Firstly, the CBAM attention mechanism was introduced between the P2 and P3 layers in the backbone layer, enhancing useful feature mapping of input features, reducing the impact of irrelevant features, and focusing on the importance of each pixel in the feature map and accordingly allocating higher weights [54]. Then, a P2 small-object detection head was added in the neck layer, enabling fast and effective identification of tiny garbage targets, thus improving the model’s small-object detection performance [55]. Finally, the FPN and PANet in the neck layer were improved by the BiFPN structure, aiding the model in determining useful weights for the comprehensive fusion of high-level and low-level features [56]. Figure 15 illustrates the overall network architecture of EcoDetect-YOLO.

### 3.5. Environmental and Parameter Settings

The versions of PyTorch and Torchvision utilized in the experiments with the YOLOv5s model were 2.0.1 and 0.15.2, respectively. Training and testing were conducted in a Windows 10 environment. The hardware configuration employed in the experiments consisted of an Intel(R) Xeon(R) Silver 4210 CPU at 2.20 GHz with 32 GB RAM and an NVIDIA GeForce RTX 3090 with 40 GB of memory. The programs were executed in a Pycharm compiler environment running on CUDA 11.8 and Python 3.8.16.

During the model training process, the proposed dataset was trained for 300 epochs. The input image size was set to 640 × 640 pixels. The SGD optimizer, based on gradient operations, was utilized for continuously adjustment of the model’s weight values through iterations. The initial learning rate was set to 0.01, with a weight decay of 0.005, and a batch size of 12 was employed. Table 1 illustrates the core parameters of the YOLOv5s model training process.

### 3.6. Model Evaluation Metrics

“Precision” or “positive predictive value” (precision, denoted as “P”) refers to the proportion of correctly classified positive samples to the total number of samples classified as positive by the classifier.

(22) $$Precision=\frac{TP}{TP+FP}.$$

“Recall” or “sensitivity” (recall, denoted as R) refers to the proportion of correctly classified positive samples to the total number of true-positive samples.

(23) $$Recall=\frac{TP}{TP+FN},$$

In Equations (22) and (23), *TP* represents the number of instances where the sample value is positive and is correctly predicted as positive, *FP* represents the number of instances where the sample value is positive but predicted as negative, and *FN* represents the number of instances where the sample value is negative but predicted as positive.

mAP is defined in Equation (25) as the approximate area enclosed by the precision–recall curve.

(24) $$AP=\int_{0}^{1}P\left(R\right)dR,$$

(25) $$mAP=\frac{\sum_{i=1}^{n}{AP}_{i}}{n},$$

Here, *mAP_0.5_* is the average precision calculated at an IOU threshold of 0.5, while *mAP_0.5:0.95_* is a more stringent comprehensive evaluation metric calculated at IOU thresholds ranging from 0.5 to 0.95.

FLOPs (floating point operations per second) are commonly used to measure model complexity. A lower FLOP value indicates a faster model execution speed, as represented by Equations (26) and (27).

(26) $$FLOPs\left(Conv\right)=\left(2\times C_{in}\times K^{2}-1\right)\times W_{out}\times H_{out}\times C_{out},$$

(27) $$FLOPs\left(Conv\right)=\left(2\times C_{in}-1\right)\times C_{out},$$

In Equations (26) and (27), *C_in_* and *C_out_* represent the input and output channels, respectively, while *K*, *H_out_*, and *W_out_* represent the size of the convolution kernel and the height and width of the output feature map, respectively.

## 4. Experimental Result and Discussion

### 4.1. Performance Evaluation

The proposed EcoDetect-YOLO model was validated using the constructed domestic waste exposure detection dataset. Experimental results, as presented in Table 2, demonstrate that the proposed EcoDetect-YOLO model achieved increases of 3.5%, 1.7%, 4.7%, and 1.0% in precision, recall, mAP_0.5_, and mAP_0.5:0.95_, respectively.

**Figure 16:**
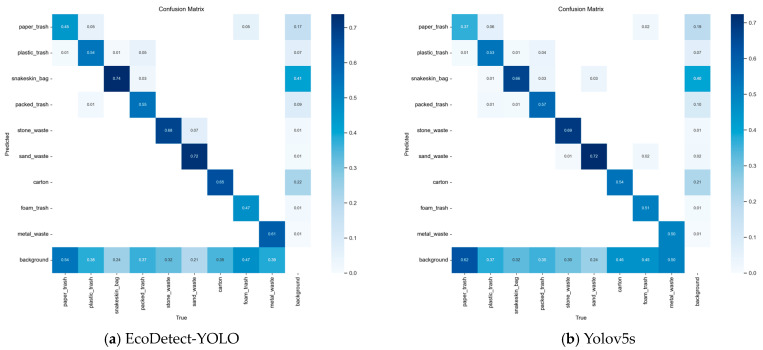
Confusion matrix of EcoDetect-YOLO and Yolov5s in the training set. (**a**) Confusion matrix of EcoDetect-YOLO. (**b**) Confusion matrix of Yolov5s.

Figure 16 presents the confusion matrix contrasting EcoDetect-YOLO and YOLOv5s, illuminating the substantial enhancements in accuracy achieved by EcoDetect-YOLO across numerous categories. Particularly noteworthy is the pronounced 11% augmentation in classification accuracy for metal waste and carton. Moreover, paper trash and snakeskin bag exhibit notable improvements of 8% in their respective classification accuracies. These findings decisively establish the superior predictive and generalization capabilities of the EcoDetect-YOLO model introduced in this study compared to the baseline YOLOv5s framework.

Although EcoDetect-YOLO demonstrated strong overall performance, its accuracy in detecting paper waste remained suboptimal, with a mAP_0.5_ of only 45%. This limitation may stem from the considerable variability and diverse shapes of paper waste, making it challenging for the model to effectively discern them. Furthermore, confusion among paper waste, plastic waste, and foam waste types was prevalent, likely due to their common white coloration and the greater distance of these wastes from the camera, complicating accurate type classification based solely on shape. Additionally, plastic waste frequently triggered misclassifications with packaging waste, possibly due to their similar shapes. The misidentification of stone waste as sand waste is also notable, possibly arising from the presence of sand waste data mixed within some stone waste samples. In summary, while further optimization is required for finer types of waste classification tasks, the model’s current performance is sufficient for practical applications.

**Figure 17:**
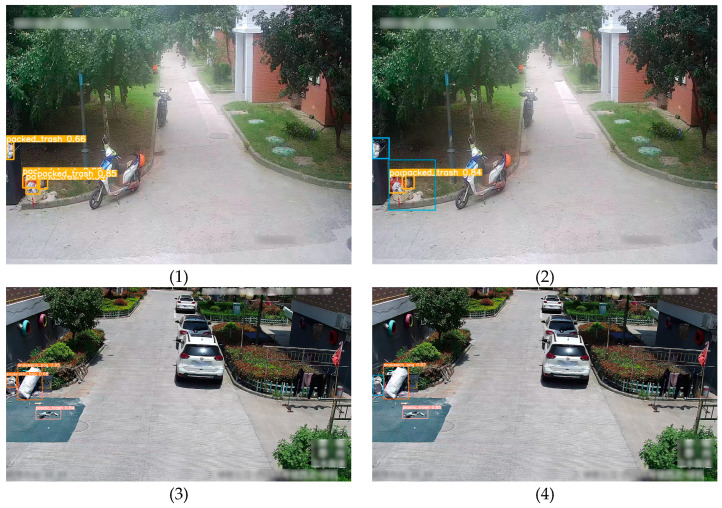

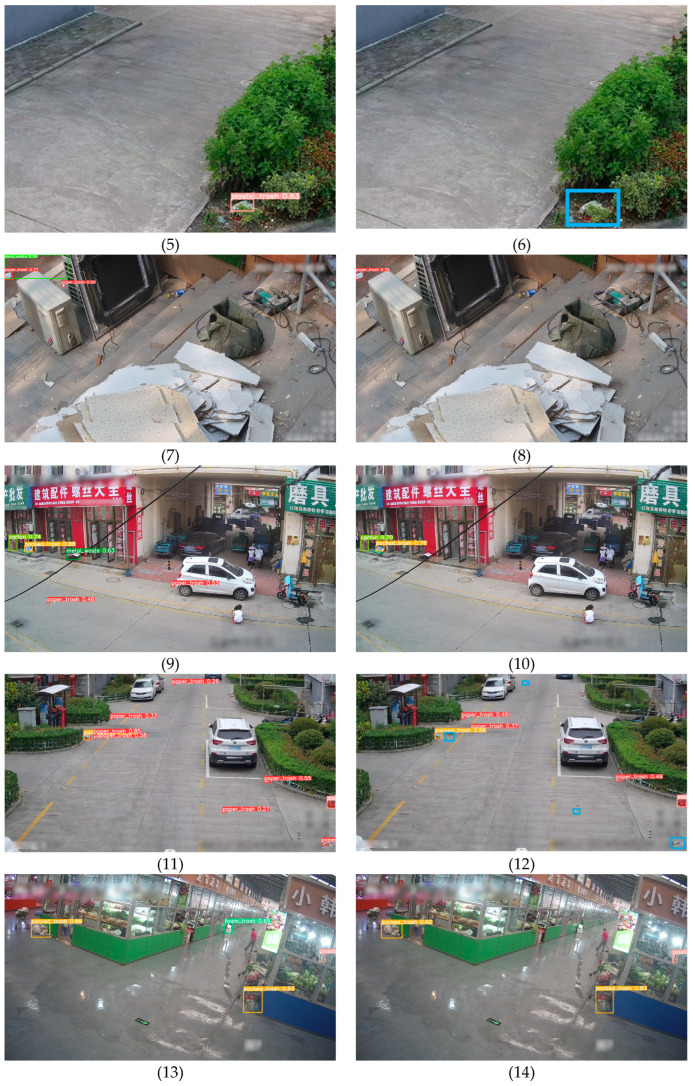

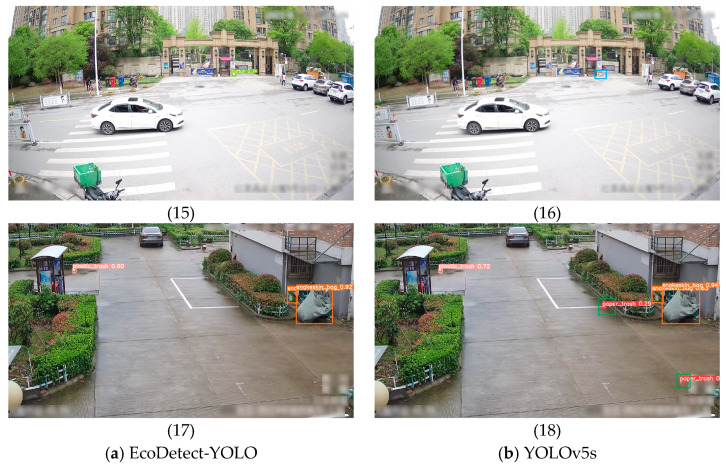
Detection results of the EcoDetect-YOLO and YOLOv5s models. (**a**) The test results of the proposed EcoDetect-YOLO model. (**b**) The test results of the baseline YOLOv5s model. Blue boxes added to the figures represent the missed detection of target samples by the model, while green boxes indicate incorrectly detected target samples.

As indicated by the results in Figure 17, the baseline YOLOv5s model tends to exhibit missed detections when the features of the target image are incomplete. For instance, in Figure 17(b2,b4,b8), the packed trash, snakeskin bag, and sand waste are located at the edges of the images, thus providing incomplete information. Similarly, the plastic trash in Figure 17(b6) is obscured by some bushes, and part of the features of the metal waste in Figure 17(b10) is obstructed by a thick black wire. Additionally, in the presence of small targets in the image, the baseline YOLOv5s model also tends to miss detections, as illustrated in Figure 17(b12,b14,b16), where the images contain relatively small paper trash, foam trash, and carton targets. In contrast, the proposed EcoDetect-YOLO model can adapt to these scenarios. Moreover, the EcoDetect-YOLO model engendered higher confidence in its detection of targets. Conversely, the baseline YOLOv5s model misidentified white parking lines as paper trash, as shown in Figure 17(b18). The comparative analysis of the results indicated that the improved EcoDetect-YOLO model, incorporating three enhancement points, effectively adapts to variations in sample sizes and feature changes, thereby enhancing the accuracy of target detection and reducing missed detection rates.

### 4.2. Analysis of Attention Mechanisms

To verify the advantages of the CBAM attention mechanism, this study conducted experimental comparisons by incorporating channel attention (CA), squeeze-and-excitation block (SE), and efficient channel attention (ECA) into the baseline YOLOv5s model. The CA attention mechanism is position sensitive and direction aware, which enhances the model’s precision in determining target positions by integrating positional and directional information of features [57]. The SE attention mechanism assigns weights to each channel based on their importance, aiming to address the issue of loss incurred during the feature extraction process of the YOLOv5s model due to varying weights assigned to each channel of the feature maps [58]. The ECA model is a lightweight channel attention module that replaces fully connected layers with 1 × 1 convolutional layers, reducing the model’s parameters [59]. This model circumvents the side effects associated with dimensionality reduction and effectively captures inter-channel interactions, thereby better extracting the essential features of the targets.

As depicted in Table 3, the YOLOv5s+CBAM achieved the highest mAP_0.5_ performance at 56.2%. Compared to YOLOv5s+CA, YOLOv5s+SE, and YOLOv5s+ECA, it improved by 1.9%, 2.4%, and 3.2%, respectively. Additionally, it achieved the best mAP_0.5:0.95_ performance at 30.4%, corresponding to improvements of 0.5%, 0.8%, and 0.6%, respectively. Moreover, it can be observed that the addition of attention models to the YOLOv5s model did not significantly alter its weights or FLOPs. Through comprehensive experimental comparisons, it was found that the CBAM attention mechanism maintains the lightweight nature of the YOLOv5s model while enhancing its accuracy.

### 4.3. Results of Ablation Experiments

This study integrated the CBAM, P2, and BiFPN modules into the YOLOv5s model in various combinations, and the results of the ablation experiments are presented in Table 4.

① The baseline YOLOv5s model yielded a precision, recall, mAP_0.5_, and mAP_0.5:0.95_ of 71.0%, 51.9%, 53.4%, and 30.1%, respectively. Additionally, it exhibited the smallest weights, FLOPs, and parameters of 14.4 MB, 15.8 G, and 6.7 MB, respectively. The FPS was the highest at 41.37 frames per second. These metrics indicate that the baseline YOLOv5s, leveraging FPN and PANet structures for multi-scale feature fusion of input images, is characterized by its lightweight features and excellent real-time garbage-object-detection capabilities. Although its detection performance is relatively modest, it meets the preliminary requirements for deployment on mobile devices for real-time complex scene garbage detection.
② After the CBAM attention module was incorporated into the YOLOv5s model, its precision, recall, mAP_0.5_, mAP_0.5:0.95_, weights, FLOPs, parameters, and FPS were 74.5%, 52.3%, 56.2%, 30.4%, 14.5 MB, 15.9 G, 6.7 MB, and 40.92 frames per second, respectively. The CBAM attention module enhances feature maps by focusing on important spatial and channel information, improving feature representation. Compared to the baseline YOLOv5s model, it achieved relative increases of 3.5%, 0.4%, 2.8%, and 0.3% in precision, recall, mAP_0.5_, and mAP_0.5:0.95_, respectively, with minor increases of 0.1 MB in weights and 0.1 G in FLOPs; parameters remained unchanged, and FPS decreased slightly by 0.45 frames per second. These performance metrics indicate that adding the CBAM module maintains the lightweight nature and computational efficiency of the YOLOv5s model, significantly enhancing detection performance. This ensures minimal additional memory burden when deployed on mobile devices, while achieving high precision in real-time detection of complex scenarios such as garbage detection.
③ After the P2 small target module was incorporated into YOLOv5s, its precision, recall, mAP_0.5_, mAP_0.5:0.95_, weights, FLOPs, parameters, and FPS were 70.2%, 56.8%, 55.4%, 30.2%, 15.3 MB, 18.6 G, 6.9 MB, and 37.95 frames per second, respectively. The P2 small target module captures additional feature information from smaller target samples, enhancing detection capabilities for these targets. Compared to the baseline YOLOv5s model, it improved recall, mAP_0.5_, and mAP_0.5:0.95_ by 4.9%, 2.0%, and 0.1% respectively. However, despite detecting more samples, it also introduced errors leading to an 0.8% decrease in precision. Furthermore, there were significant increases in weights, FLOPs, and parameters, with increments of 0.9 MB, 2.8 GB, and 0.2 MB respectively. FPS notably decreased by 3.42 frames per second. This indicates that adding the P2 detection layer introduces higher computational complexity and parameters. While it enhances the detection capability of smaller target samples, it compromises model accuracy and frame rate. Consequently, when deployed on mobile devices, although the increase in memory usage is modest relative to modern hardware and FPS remains sufficient for real-time performance, the decreased detection accuracy limits its suitability primarily to garbage-object-detection systems with lower precision requirements.
④ After the improved BiFPN structure was incorporated into the YOLOv5s model, its precision, recall, mAP_0.5_, mAP_0.5:0.95_, weights, FLOPs, parameters, and FPS were 71.8%, 51.9%, 54.1%, 29.4%, 14.7 MB, 16.5 G, 6.9 MB, and 39.76 frames per second, respectively. It can be observed that relative to the baseline YOLOv5s model using the FPN and PANet structure, the performance was improved, with an increase in precision and mAP_0.5_ of 0.8% and 0.7%, respectively. This indicates that the BiFPN structure effectively handles information from multi-scale features during feature fusion and optimizes feature representation capability. Additionally, it introduces extra model complexity and computational overhead compared to the FPN and PANet structure, with increases in weights and parameters of 0.3 MB and 0.2 MB, respectively. Inference-time FLOPs also were also increased by 0.7 G, while FPS decreased by 1.61 frames per second. Although the introduction of the BiFPN structure slightly increases computational overhead and model complexity, its impact on FPS reduction and memory increase on modern hardware remains minimal. By enhancing feature fusion capability, BiFPN improves the model’s detection performance, making it feasible and practical for real-time garbage-object-detection models requiring higher detection accuracy.
⑤ After the CBAM and BiFPN modules were incorporated into the YOLOv5s model, its precision, recall, mAP_0.5_, mAP_0.5:0.95_, weights, FLOPs, parameters, and FPS were 69.1%, 51.6%, 53.3%, 29.2%, 14.7 MB, 16.6 G, 6.9 MB, and 39.58 frames per second, respectively. Due to the overlapping functionalities of enhancing features and adjusting weights within both CBAM and BiFPN modules, there is a conflict in their combined effectiveness. During module training, CBAM’s handling of feature map information through channel and spatial attention affects the multi-scale fusion performance of BiFPN, resulting in a combined effect that does not improve and instead decreases performance. Relative to the YOLOv5s model with only the BiFPN module added, the precision, recall, mAP_0.5_, and mAP_0.5:0.95_ decreased by 2.7%, 0.3%, 0.8%, and 0.2%, respectively, when CBAM was also included. Additionally, there was a slight increase in FLOPs of 0.1 GB, while weights and parameters remained unchanged. FPS decreased by 0.18 frames per second. This indicates that adding CBAM to the original BiFPN module, while not significantly impacting computational complexity or FPS, has a considerably negative effect on the model’s detection accuracy. In some cases, the overall detection performance of the combined model was inferior to the original YOLOv5s model. Therefore, in practical deployment scenarios, this combined configuration is generally not considered favorable. Instead, it may be preferable to choose the YOLOv5s model alone or to separately add the CBAM attention module and BiFPN module to enhance model usability effectively while minimizing costs.
⑥ After the P2 and BiFPN modules were incorporated into the YOLOv5s model, its precision, recall, mAP_0.5_, mAP_0.5:0.95_, weights, FLOPs, parameters, and FPS were 76.9%, 51.5%, 56.5%, 30.6%, 15.4 MB, 19.2 G, 6.9 MB, and 37.26 frames per second, respectively. During training, the model enhances its ability to capture and detect small target samples through the P2 detection layer and optimizes multi-scale feature fusion via BiFPN. Compared to the baseline YOLOv5s model, although the model produced significant increases in weights and FLOPs of 1 MB and 3.4 G, respectively, parameters increased by 0.2 MB, and FPS decreased notably by 4.11 frames per second; more importantly, precision, mAP_0.5_, and mAP_0.5:0.95_ improved by 5.9%, 3.1%, and 0.5%, respectively. In comparison to the YOLOv5s models with only the P2 detection layer or only the BiFPN module added, mAP_0.5_ increased by 1.1% and 2.4% respectively, with precision increasing by 6.7% and 5.1%, respectively. By observing these performance metrics, it is evident that the YOLOv5s model with the P2 and BiFPN modules achieves significantly improved detection performance despite the overall increase in model complexity and computational overhead. As it achieved the highest precision level and maintained sufficient FPS for real-time requirements, deploying this model on mobile devices may only necessitate additional hardware memory costs, enabling effective real-time garbage detection in complex environments with high detection accuracy demands.
⑦ After the CBAM and P2 detection layer were incorporated into the YOLOv5s model, its precision, recall, mAP_0.5_, mAP_0.5:0.95_, weights, FLOPs, parameters, and FPS were 73.0%, 55.5%, 56.4%, 30.3%, 15.4 MB, 18.8 G, 6.9 MB, and 37.63 frames per second, respectively. During model training, CBAM enhances important regions and channels within feature maps to improve feature representation. Subsequently, the P2 detection layer refines this process, enhancing detection capabilities for small target samples. Compared to the YOLOv5s model with only the P2 detection layer added, the precision, mAP_0.5_, and mAP_0.5:0.95_ improved by 2.8%, 1.0%, and 0.1%, respectively. Although there were slight increases in weights and FLOPs of 0.1 MB and 0.1 G, respectively, parameters remained unchanged, and FPS slightly decreased by 0.32 frames per second. These parameter changes indicate that adding the CBAM module on top of the P2 detection layer allows the model to maintain a similar model size and computational overhead as the YOLOv5s model with only the P2 detection layer while addressing the shortcomings in detection accuracy. This adaptation enables the model to fit into garbage-detection systems requiring high precision and real-time capabilities.
⑧ Due to partial conflicts in functionality between the CBAM and BiFPN modules leading to decreased model performance, the addition of a P2 detection layer between them serves as a buffer. Initially, the CBAM attention mechanism enhances features, which are then further refined by the P2 detection layer. Subsequently, BiFPN performs weighted multi-scale feature fusion. This sequence effectively mitigates conflicts in information processing that arise from combining CBAM and BiFPN, thereby significantly improving model performance.

In comparison to the baseline YOLOv5s model, the EcoDetect-YOLO model, after integrating three modules, demonstrated the highest weights, FLOPs, and parameters of 15.7 MB, 20 G, and 7.0 MB, respectively, across all combined strategies, with an FPS of 36.56, a decrease of 4.81 frames per second. Despite this, the model retained its capability for lightweight and real-time detection. Importantly, the model achieved optimal detection accuracy, with improvements in precision, recall, mAP_0.5_, and mAP_0.5:0.95_ of 3.5%, 1.7%, 4.7%, and 1.0%, respectively. Overall, the combination of these three modules significantly enhances the EcoDetect-YOLO model across all metrics, enabling it to be deployed on mobile devices for highly accurate garbage detection in complex environments in real-time.

### 4.4. Comparison of Different Methods

This study selected single-stage object detection models SSD, YOLOv3, YOLOv3-tiny, YOLOv8s, YOLOv8l, and YOLOv5 models with variations in width and depth to derive the YOLOv5m and YOLOv5l models. Additionally, models structurally similar to those used in this study and state-of-the-art waste detection models, namely GCC-YOLO, Waste-YOLO, and YOLOv5s-OCDS, were incorporated. The results of models’ training under identical experimental parameters are presented in Table 5.

According to the results in Table 5, compared with the single-stage object detection model SSD, the proposed EcoDetect-YOLO model maintains significantly improved mAP_0.5_ and FPS with lower model size and parameters. The baseline YOLOv5s model, similar in network structure to YOLOv3, yields inferior comprehensive performance due to its lower model size and parameters compared to YOLOv3. In contrast, while achieving higher mAP_0.5_ than YOLOv3, the EcoDetect-YOLO model exhibits lower model size and faster detection speed.

YOLOv3-tiny, a simplified version of YOLOv3 with similar network depth and width, shows faster detection speed and similar model size and parameters compared to EcoDetect-YOLO. However, its mAP_0.5_ was below 50%, indicating slightly lower detection accuracy. YOLOv5m and YOLOv5l are models within the YOLOv5 series that vary in network width and depth. By increasing model size and parameters compared to the base YOLOv5s model, both YOLOv5m and YOLOv5l show significantly improved performance. However, they achieve lower mAP_0.5_ and FPS relative to the EcoDetect-YOLO model, while also having larger model size and more parameters. 

YOLOv8 models, as advanced models within the YOLO series, are represented by YOLOv8s and YOLOv8l in this study. YOLOv8s demonstrated a slightly faster detection speed compared to EcoDetect-YOLO, while its corresponding mAP_0.5_ was comparatively lower, and it featured a larger model size and more parameters. YOLOv8l exhibits lower mAP_0.5_ and detection speed compared to EcoDetect-YOLO, with significantly higher model size and parameters.

In the GCC-YOLO model, the combination of YOLOv5s+C3GC+P2+BiFPN was chosen for comparison with our EcoDetect-YOLO model. GCC-YOLO enhances the YOLOv5s model by incorporating a global context (GC) attention module combined with the C3 module, known as the C3GC module, which suppresses background noise and improves feature extraction capabilities. Similarly, it also employs modules P2 and BiFPN, which are identical to those in our study. Our proposed EcoDetect-YOLO model outperformed GCC-YOLO in terms of parameters, FPS, and mAP_0.5_. However, it exhibited slightly higher FLOPs. This is because while the C3GC module in GCC-YOLO focuses on capturing global contextual features, the EcoDetect-YOLO model introduced in our study utilizes the CBAM module at the backbone level. CBAM enhances feature representation by incorporating attention mechanisms across both channel and spatial dimensions. This dual-dimensional attention mechanism proves advantageous in complex backgrounds, allowing the model to better focus on key features and thereby improving overall performance.

Relative to the Waste-YOLO model, an advanced waste detection model that shares a similar combination with the EcoDetect-YOLO model, differs primarily in the replacement of the P2 module with C3CA (coordinate attention integrated into C3 module). Although the Waste-YOLO model exhibited superior FPS, model size, and parameters over the EcoDetect-YOLO model, it overlooked scenarios involving numerous small-target samples. Furthermore, conflicts between attention mechanisms and BiFPN modules led to a decline in overall module performance.

The YOLOv5s-OCDS is an advanced garbage detection model. Compared to YOLOv5s-OCDS, the EcoDetect-YOLO model proposed in this study has a smaller model size and fewer parameters, while achieving better mAP_0.5_ and FPS performance. 

In the results analysis, the EcoDetect-YOLO model proposed in this study achieved the highest mAP_0.5_ among all models, surpassing SSD, YOLOv3, YOLOv3-tiny, YOLOv5s, YOLOv5m, YOLOv5l, YOLOv8s, YOLOv8l, GCC-YOLO, Waste-YOLO, and YOLOv5s-OCDS by 3.7%, 0.6%, 12.4%, 4.7%, 2.9%, 1.6%, 2.7%, 1.3%, 1.1%, 4.5%, and 4.0%, respectively. Additionally, achieving an FPS of 36.56, the model maintained a model size, parameters, and FLOPs close to those of the baseline YOLOv5s model, thereby meeting the requirements for accurate and real-time detection of conventional waste exposure in real-world scenarios and promoting advancements in related waste detection model technologies.

**Figure 18:**
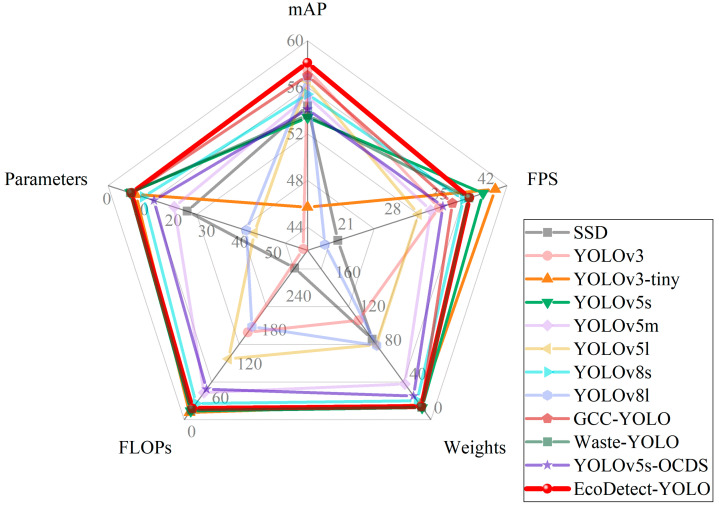
Performance comparison of the different detection algorithms.

From Figure 18, it is evident that the EcoDetect-YOLO model achieved the highest mean average precision (mAP) while maintaining relatively low levels of parameters, weights, and FLOG, three indicators measuring model size and complexity. This underscores the superior performance of the EcoDetect-YOLO model in detection accuracy compared to the other eleven models and its ability to simultaneously retain low complexity and lightweight characteristics. Of particular note is that the proposed EcoDetect-YOLO model exhibited a negligible decrease in detection rate compared to the YOLOv5s model, with a mere 2.01 decrease in frames per second (FPS). Overall, the EcoDetect-YOLO model demonstrates excellent overall performance, facilitating precise and efficient real-time detection of domestic waste in intricate environmental landscapes.

### 4.5. Robustness Testing

**Figure 19:**
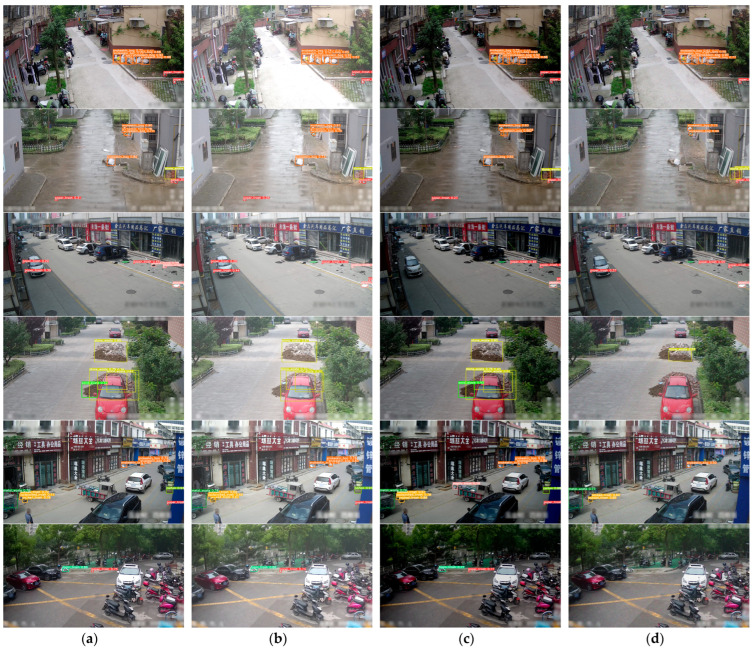
Robustness test results. (**a**) The original image. (**b**) The image with increased brightness, (**c**) The image with decreased brightness. (**d**) The image with added Gaussian noise.

Due to various objective environmental factors encountered during camera capture in real-world scenarios, such as overexposure, dim lighting conditions, and blurry images, the evaluation of the adaptability of the proposed EcoDetect-YOLO model to such scenarios is crucial. In this study, three processing methods, including brightness enhancement, brightness reduction, and addition of Gaussian noise, were applied to the test set images, and the experimental results obtained are illustrated in Figure 19.

As depicted in Figure 19, under conditions of increased brightness, decreased brightness, and added Gaussian noise, nine types of garbage, including snakeskin bag, paper trash, stone waste, packed trash, and plastic trash, could be accurately detected. However, there were also instances of both missed detections and false alarms:

(1) As illustrated in the first to third rows, paper trash, plastic trash, and snakeskin bags originally exhibit white characteristics. In situations of dim or blurry image lighting, the overall color of the samples tend to be darker, resulting in detection difficulties for the model.
(2) As shown in the third to fourth rows, packed trash, and stone waste are primarily gathered and piled up. When the image becomes blurry, its features become more complex, making it difficult for the model to collect sample features during the detection process, thus leading to missed detections.
(3) As indicated in the fourth row, sand waste, due to its dark-colored and clustered characteristics, can be detected when decreasing brightness but may result in missed detections under conditions of increased brightness and blurriness.
(4) As shown in the fourth to fifth rows, carton and foam trash may produce missed detections when appearing small in the image due to image blurriness.
(5) As depicted in the third row, under conditions of decreased brightness and blurriness, the model may produce false positives, detecting paper trash as plastic trash.

Despite the model’s tendency to miss detections in blurry images, it demonstrated its capability for concurrent detection overall. The final mAP_0.5_ result of the experiment still exceeded 50%. In summary, the improved EcoDetect-YOLO model exhibits good robustness under conditions of increased brightness, decreased brightness, and added Gaussian noise.

## 5. Conclusions

Due to challenges associated with small sample sizes, intricate environmental landscapes, and multi-scale issues in garbage captured by cameras, the real-time detection of waste exposure in regular garbage dumps and residential community streets is inherently challenging. Addressing this issue, this paper proposes an improved object detection model, EcoDetect-YOLO, based on the YOLOv5s model, combined with a dataset of domestic waste in intricate road environments. The analysis of experimental results revealed that the baseline YOLOv5s model achieved a mAP_0.5_ and mAP_0.5:0.95_ of 53.4% and 30.1%, respectively. Three improvements over the YOLOv5s model yield the following observations: (1) The CBAM attention mechanism enhances the focus on important features in both channel and spatial dimensions, effectively improving the model’s performance. Compared to the baseline YOLOv5s model, it achieved an increase of 2.8% and 0.3% in mAP_0.5_ and mAP_0.5:0.95_, respectively, while maintaining lightweight advantages. (2) Adding a P2 small-object detection head improved the detection of small objects, enhancing the model’s performance. Compared to YOLOv5s, it achieved an increase of 2.0% and 0.1% in mAP_0.5_ and mAP_0.5:0.95_, respectively. (3) BiFPN, an improvement based on the FPN and PANet structures, facilitates rapid fusion of multi-scale features, improving feature fusion efficiency and small-object detection. Compared to the baseline YOLOv5s model, it achieved a 0.7% increase in mAP_0.5_. By integrating the CBAM attention mechanism, P2 small-object detection layer, and BiFPN, the proposed EcoDetect-YOLO model achieved a mAP_0.5_ and mAP_0.5:0.95_ of 58.1% and 31.1%, respectively, surpassing the YOLOv5s-based model by 4.7% and 1.0%, respectively. The model size is 15.7 MB, with a detection speed of 39.36 frames per second (FPS). Additionally, the EcoDetect-YOLO model exhibited good performance and robustness even in extreme scenarios.

In summary, the proposed EcoDetect-YOLO model offers high detection accuracy, fast detection speed, and a relatively small model size, making it suitable for real-time detection and control of waste exposure in regular garbage dumps and residential community streets, especially when deployed on mobile devices.

## Figures and Tables

**Table 1:** Training parameters of the YOLOv5s Model.

Parameter Name	Parameter Value
Initial learning rate	0.01
Momentum	0.937
Weight decay	0.0005
Epochs	300
Batch size	12
Noautoanchor	False
Input image size	640
Optimizer	SGD

**Table 2:** Comparison of results between the YOLOv5s model and the EcoDetect-YOLO model.

Model	P	R	mAP_0.5_ (%)	mAP_0.5:0.95_ (%)	Weight (MB)
YOLOv5s	71.0	51.9	53.4	30.1	14.4
EcoDetect-YOLO	74.5	53.6	58.1	31.1	15.7

**Table 3:** Comparison of the attention mechanism results.

Model	mAP_0.5_ (%)	mAP_0.5:0.95_ (%)	Weights (MB)	FLOPs (G)
YOLOv5s	53.4	30.1	14.4	15.8
YOLOv5s+CA	54.3	29.9	14.5	15.9
YOLOv5s+SE	53.8	29.6	14.5	15.9
YOLOv5s+ECA	53.0	29.8	14.4	15.8
YOLOv5s+CBAM	56.2	30.4	14.5	15.9

**Table 4:** Results of ablation experiments.

Number	CBAM	P2	BiFPN	P	R	mAP_0.5_	mAP_0.5:0.95_	Weight (MB)	FLOP (G)	Parameter (MB)	FPS
①	-	-	-	71.0	51.9	53.4	30.1	14.4	15.8	6.7	41.37
②	T	-	-	74.5	52.3	56.2	30.4	14.5	15.9	6.7	40.92
③	-	T	-	70.2	56.8	55.4	30.2	15.3	18.6	6.9	37.95
④	-	-	T	71.8	51.9	54.1	29.4	14.7	16.5	6.9	39.76
⑤	T	-	T	69.1	51.6	53.3	29.2	14.7	16.6	6.9	39.58
⑥	-	T	T	76.9	51.5	56.5	30.6	15.4	19.2	6.9	37.26
⑦	T	T	-	73.0	55.5	56.4	30.3	15.4	18.8	6.9	37.63
⑧	T	T	T	74.5	53.6	58.1	31.1	15.7	20.0	7.0	36.56

**Table 5:** The experimental results of the different models.

Model	mAP_0.5_	FPS	Weight (MB)	FLOP (G)	Parameter (MB)
SSD	54.4	19.56	95.1	268.5	23.8
YOLOv3	57.5	34.75	117.3	154.7	58.7
YOLOv3-tiny	45.7	43.23	17.5	12.9	8.3
YOLOv5s	53.4	41.37	14.4	15.8	6.7
YOLOv5m	55.2	33.54	42.2	48.0	19.9
YOLOv5l	56.5	31.68	88.58	107.8	44.0
YOLOv8s	55.4	38.45	22.4	28.8	10.6
YOLOv8l	56.8	17.61	87.7	164.9	41.6
GCC-YOLO [60]	57.0	36.79	15.3	19.8	7.2
Waste-YOLO [61]	53.6	39.27	14.7	16.7	6.71
YOLOv5s-OCDS [62]	54.1	35.35	28.0	54.2	13.81
Our EcoDetect-YOLO	58.1	39.36	15.7	20.0	7.0

## Data Availability

The datasets used and analyzed during the current study are available from the corresponding author upon reasonable request.
